# The Next Phase of 3D Bioprinting: AI-Native Systems—A Narrative Review

**DOI:** 10.3390/jfb17070319

**Published:** 2026-07-03

**Authors:** Nebojša Zdravković, Mateja Zdravković, Marko N. Živanović

**Affiliations:** 1 Department of Medical Statistics and Informatics, Faculty of Medical Sciences, University of Kragujevac, Svetozara Markovića 69, 34000 Kragujevac, Serbia; nzdravkovic@fmn.kg.ac.rs; 2 Institute for Information Technologies Kragujevac, University of Kragujevac, Liceja Kneževine Srbije 1A, 34000 Kragujevac, Serbia; marko.zivanovic@uni.kg.ac.rs

**Keywords:** AI-native bioprinting, 3D bioprinting, biological intelligence, closed-loop systems, machine learning

## Abstract

Three-dimensional (3D) bioprinting has reached a complexity limit where empirical, parameter-by-parameter optimization no longer scales. The dominant mode of artificial intelligence (AI) integration remains AI-augmented, where AI is treated as an analytical addition to a conventional pipeline. We argue that the field is approaching a discontinuous transition towards AI-native bioprinting, in which AI represents the operational layer of system intelligence, not an ancillary tool. A systematic analysis of 365 publications on the intersection of bioprinting and AI (2015–2026), performed through 18 queries organized by the four search axes of the PubMed database, shows that the intersection grew 136 times during the decade, with an acceleration of 3.16 times only between 2024 and 2025. Mapping the publications to the six functional domains reveals a marked asymmetry: clinical translation counts 154 papers, while cell viability prediction—the biological foundation that every closed-loop system requires—counts only three. We define AI-native bioprinting as a system architecture that combines continuous learning, multi-modal sensing fused through visual, mechanical and biological signals, and biologically closed control loops. We present a conceptual shift from printing accuracy to biological intelligence as a success criterion. The transition requires open datasets, consensus biological metrics, inter-laboratory validation, and early regulatory engagement.

## 1. Introduction

### 1.1. Bioprinting as a Mature, but Already Over-Extended Field

Three-dimensional bioprinting (3D bioprinting) in the last decade has gone from demonstration experiments with hydrogels to a platform capable of fabricating human tissues in clinical dimensions [1,2,3]. A systematic analysis of the PubMed literature for the period 2015–2026 (detailed methodology in Section 1.4 and Supplementary Section S1) shows that the field published over 10,000 papers during that period, with a 10.4-fold increase in ten-year output (158 publications in 2015 vs. 1648 in 2025). This growth is not linear: it is followed by a jump in the complexity of the process itself. Today, techniques such as vat photopolymerization, extrusion-based bioprinting, inkjet bioprinting and the FRESH (freeform reversible embedding of suspended hydrogels) approach together form a modular repertoire [1,4], and the class of bioinks includes GelMA, alginate, collagen, peptide-modified gellan polymers and hybrid composite systems with controlled crosslinking chemistry [2,5,6].

What is particularly important is that the field has expanded across too many control parameters. Already in 2016, an integrated tissue–organ printer (ITOP) [3] combined cell-laden hydrogels with biodegradable polymers and sacrificial hydrogels, using clinical image diagnostics as input to a CAD model and, further, to a print nozzle control program. Three years later, the FRESH platform extended the resolution scale from capillaries to the entire ventricular system, demonstrating synchronized contractions and propagation of action potentials in cardiac constructs [4]. In other words, the number of parameters influencing the outcome (bioink properties, nozzle settings, crosslinking profile, cell type, spatial arrangement, post-printing maturation) has become greater than what manual optimization by trial-and-error can cover.

### 1.2. AI Is Growing in Parallel—But the Intersection Is Growing Faster

**Figure 1:**
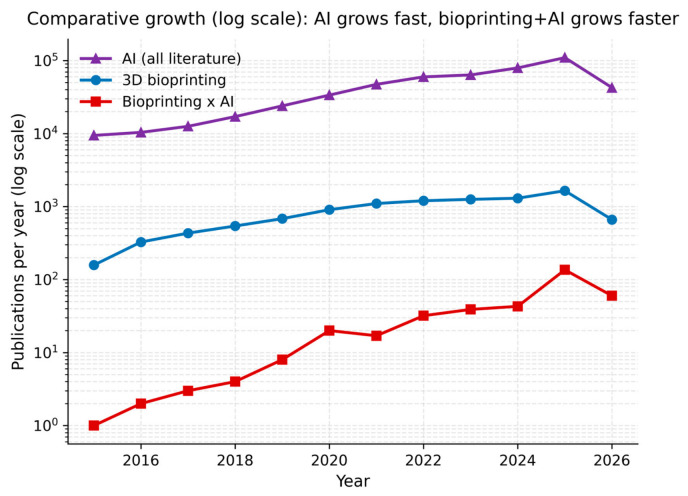
Logarithmic representation of the annual production of publications in three populations: all AI literature (purple), all 3D bioprinting literature (blue) and their intersection (red), for the period 2015–2026. The logarithmic scale reveals that the cross-section grows with a different slope compared to individual fields, with a sharp acceleration in the 2024–2025 period. A linear version of this distribution is given in Figure S1 of the Supplementary Material. A log-linear fit over 2015–2025 confirms this visually distinct slope: the intersection grows at approximately 57% per year (R^2^ = 0.97), compared with approximately 23% for bioprinting and approximately 30% for AI overall—a 2.2-fold steeper trajectory than the bioprinting field itself (full per-year counts in Supplementary Table S1).

**Figure 2:**
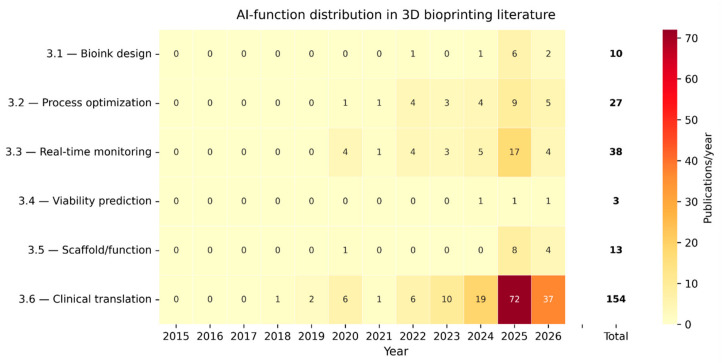
Distribution of publications at the intersection of 3D bioprinting and AI by six functional categories (AIF) and for the years 2015–2026. The heatmap shows a marked asymmetry: clinical translation (Section 3.6) dominates with 154 publications, while cell viability prediction (Section 3.4) counts only 3. The rightmost column (“Total”) gives the per-row sum across all years, so that each category’s full-decade count can be read directly without summing individual cells. Aggregate figures for the entire ten-year period are also given in Figure S3 of the Supplementary Material.

In the same time frame, the AI literature grew 11.6 times (from 9438 publications in 2015 to 109,673 in 2025). By themselves, these numbers reflect a general trend and are not specific to biomedicine. What is new and fundamentally important is the acceleration of the intersection: the literature at the intersection of 3D bioprinting and AI has grown 136 times over the same period—from 1 publication in 2015 to 136 in 2025. Between 2024 and 2025 alone, the number of cross-sectional papers tripled (43 → 136, multiplier of 3.16-fold). Figure 1 shows these three growths on a logarithmic scale, which clearly shows the different slope of the intercept curve in relation to the slopes of the individual fields. The PubMed search was executed on 20 April 2026; the 2026 column therefore reflects a partial year (n = 663 bioprinting, n = 42,576 AI, n = 60 at the intersection at the cutoff date), and no projection or extrapolation was applied. To avoid bias from this incomplete year, all fold-change and log-linear regression statistics reported here are computed on the 2015–2025 window, while 2026 counts are retained in Figure 1 and Figure 2, and Supplementary Table S1 for transparency.

This is not just the parallel growth of the two fields—it is their active merging. While Polymers for 3D Printing and Customized Additive Manufacturing [1] from 2017, the most-cited work on materials for 3D printing, focused exclusively on polymer chemistry, Progress and Opportunities for Machine Learning in Materials and Processes of Additive Manufacturing [7] from 2024 explicitly positions ML as an integrative axis that connects quality control, process optimization, design optimization, microstructure analysis and material formulation in a unified framework. The 7 years between these two papers coincide with a fundamental shift in how the field is conceptualized—from a “set of independent techniques” to a “prediction system with an AI layer”.

Concrete examples from the past two years illustrate this convergence. *Cell Stem Cell* [8] in 2024 demonstrates 3D bioprinting of human neural tissues with functional connections, where printed neural progenitors form cortical–striatal projections and spontaneous synaptic currents—the kind of functional validation that requires AI-assisted analysis of calcium flux and synaptic response. *Advanced Materials* [9] predicts 4D bioprinting as the next phase in 2024, but explicitly highlights (machine learning-based) modeling approaches to predict the structure–property and design-shape transformation relationships as a key technological prerequisite. *Advanced Materials* [10] in 2025 (Emerging Bioprinting for Wound Healing) interdisciplinarily positions 4D bioprinting, artificial intelligence-assisted bioprinting, and in situ bioprinting as three forces that together define the next wave in clinical application. In situ bioprinting, the third of these forces, has already moved from concept to working hardware: Zhou et al. [11] demonstrated a ferromagnetic soft catheter robot that performs minimally invasive, magnetically actuated bioprinting on internal organs in vivo, depositing a conductive hydrogel onto the liver of a living rat through a millimeter-scale incision, an operation whose accuracy the authors explicitly tie to closed-loop control with real-time intraoperative imaging, the very feedback layer that AI is positioned to supply.

### 1.3. The Field Is Functionally Fragmented—And That Is the Reason for This Review

Although the cross-section is quantitatively significant (365 papers in our analysis), it is functionally uneven. Mapping the papers by six functional domains of AI in the bioprinting pipeline (bioink design, process optimization, real-time monitoring, viability prediction, scaffold–function mapping, clinical translation) reveals a sharp asymmetry. Clinical translation dominates with 154 publications, while cell viability prediction—the functional key point of the entire technology—counts only 3 publications for the entire decade. Process optimization (27), real-time monitoring (38) and scaffold–function (13) occupy middle positions; bioink design (10) remains modestly represented (the complete distribution for all six AIF categories is shown in Figure S3, and for all 18 queries in Table S2 of the Supplementary Material).

This asymmetry is not random and is not noise. It shows that the field moved “downstream-first, upstream-later”, i.e., from clinical applications to basic biological predictions, which means that the basic science layer (prediction of outcomes at the cellular level) lags behind application. From a systems engineering perspective, this is the reverse order of what would be expected in a mature field. A systemic review that maps where AI enters the pipeline, what are the holes and what are the places of saturation becomes necessary right now, while the field is not yet cemented.

### 1.4. Search Strategy and Study Selection

The corpus underlying this review was assembled through a reproducible four-axis PubMed search executed via the NCBI Entrez API. The four axes are: 3D bioprinting (BIO; 15 Title/Abstract terms unioned with the MeSH descriptor “Bioprinting”); AI function in the bioprinting pipeline (AIF; six sub-axes—bioink design, process optimization, real-time monitoring, viability prediction, scaffold–function mapping, clinical translation—with 7–12 terms each); AI method (AIM; general machine learning, deep learning, computer vision, generative models, reinforcement and Bayesian methods, digital twin); and target tissue or application (APP; bone, cartilage, skin, vascular, neural, organoid/tumor, liver, cornea, cardiac).

Eighteen queries were constructed by combining these axes: three baseline queries (BIO, AIM, and their direct intersection BIO × AIM), six BIO × AIM × AIF intersections, and nine BIO × AIM × APP intersections. Each axis block joins Title/Abstract terms with the corresponding MeSH descriptors using Boolean OR; axis blocks are joined by AND; and every query is restricted to the publication-type filter (review [Publication Type] OR journal article [Publication Type]). No explicit [Language] filter is applied; non-English work is therefore under-represented to the extent that PubMed indexing under-represents it. The temporal window is 2015 to the data-freeze date of 28 April 2026; the year 2026 is therefore partial and is excluded from fold-change and log-linear regression calculations (Figure 1, Supplementary Table S1). The complete Boolean strings for all eighteen queries are reproduced verbatim in queries_used.txt in the reproducibility repository and listed in Supplementary Section S1.

The full pipeline (search, citation enrichment via OpenAlex, deduplication, and per-section reading-list construction) is released as open-source code at the repository cited in Supplementary Section S1.6, with all configuration files and the executable scripts. No manual screening or post hoc exclusion was applied: all PMIDs returned by the eighteen queries entered the corpus. Deduplication across queries operates on DOI (with PMID fallback when DOI is unavailable). A complementary unique-PMID overlap analysis across the six AIF sub-axes (set unions, intersections, and pair-wise overlaps) is reported in Supplementary Section S5 and Table S4. Within each query, results are kept across years and counted by publication-date year; per-year counts in Figure 1 and Figure 2, and Supplementary Table S1 reflect this per-year aggregation, in which a small number of PMIDs (~5%) with multi-year publication-date entries in PubMed can be counted under more than one year.

Two derived subsets are used in this manuscript and should be distinguished. The full corpus consists of 365 publications at the BIO × AIM intersection (data-freeze count of 28 April 2026), which forms the quantitative base for Figure 1 and Figure 2, and the functional mapping in Section 3. A top-cited subset of 78 unique publications (top 5 by all-time citations and top 5 by citations-per-year per query, deduplicated across the 17 intersection-related queries) forms the analytical base for Section 4 (Table 1) and provides the algorithmic detail that the long tail of the corpus does not consistently report; this subset is regenerated by the fetch_abstracts.py script and stored as abstracts_for_writing.json. A separate, broader master_unique.csv (184 publications, top 20 by all-time citations per query) is provided for exploratory use in the reproducibility repository.

### 1.5. The Central Thesis of This Review

The central thesis of this review, which follows from the analysis above, is straightforward:

AI should be viewed not as an auxiliary analytical tool, but as the operational intelligence layer of next-generation 3D bioprinting.

To anchor the architectural argument that runs through the manuscript, two terms are introduced here in their working form and developed in full in Section 7.1. AI-augmented bioprinting denotes a conventional workflow—bioink, printer, cell culture, and post-printing assay—into which AI components are inserted at specific points (parameter optimization, image-based monitoring, post hoc outcome prediction) without altering the workflow’s structural architecture; the AI layer is replaceable without redesigning the system. AI-native bioprinting denotes a workflow in which the AI layer is an architectural prerequisite rather than an add-on: real-time multi-modal sensing, continuous learning from every print, biological signals in the control loop, and explainable human supervision are designed into the system from the outset, and removing the AI layer collapses the workflow rather than degrading it. The distinction is not strictly binary but a spectrum along these design dimensions; existing systems sit at different points along it depending on how many of the four components are architecturally integrated rather than retrofitted. Section 7.2 develops the four components, and the An Illustrative AI-Native Workflow Section grounds the distinction in a concrete worked example.

This review follows the thesis through five pillars: (i) the nature of the complexity that has driven the field beyond empirical optimization (Section 2); (ii) a systematic map of the points at which AI enters the bioprinting pipeline (Section 3); (iii) AI methods mapped to functions, not algorithms (Section 4); (iv) conceptual shift from printing accuracy to biological intelligence (Section 5); (v) current limitations and the transition to AI-native bioprinting as the next phase of the field (Section 6 and Section 7).

## 2. The Problem of Complexity: Why Bioprinting Has Outgrown Empirical Optimization

### 2.1. A High-Dimensional, Non-Linear Parameter Space

Bioprinting is not a monolithic technique, but a workflow with a decision-making cascade, where each step opens up a new space of parameters. Ligon et al. [1], in their overview of polymers for additive manufacturing, list, only at the level of extrusion and photocrosslinking systems, more than ten classes of polymers (thermoplastics, thermosets, elastomers, hydrogels, functional polymers, polymer blends, composites, biological systems) and even more classes of process variables (build speed, accuracy, surface finish, stability, mechanical properties, porosity). When this space is restricted to the bioprinting sub-area, where the polymer matrix must be combined with living cells, the number of relevant axes increases, not decreases.

What makes this space operationally more difficult than the classic engineering problem is its non-linearity. Yue et al. [2], describing GelMA hydrogels, explicitly state that the mechanical properties depend on the degree of methacryloylation, polymer concentration, and intensity and time of exposure to UV light, as well as on the interaction with the cells that are crosslinked into the material, where none of these axes acts additively on the others. Mandrycky et al. [5] summarize this complexity explicitly: current bioprinting approaches still have “technical challenges in terms of high-resolution cell deposition, controlled cell distributions, vascularization and innervation within complex 3D tissues”. In other words, the problem is not one of these dimensions individually, but one of their simultaneous balancing.

The consequence is that empirical optimization becomes expensive and unreliable. Optimizing one axis (e.g., printability by adjusting bioink viscosity) often degrades another axis (cell viability under increased shear stress in the nozzle). Classical Design of Experiments (DoE) methodologies can capture dual interactions, but they quickly choke in the space of 8–12 simultaneous axes, which is a realistic order of magnitude for a bioink + printer + cell line system.

### 2.2. Biological Variability as a Systemic Problem

Non-linearity is difficult, but engineering can be served by better sampling. A significant qualitative leap comes with biological variability, which in bioprinting is not noise, but a structural element of the problem.

The ITOP system of Kang et al. [3] from 2016 illustrates this dramatically: an integrated printer is technically able to fabricate a mandible, calvarial bone, cartilage and skeletal muscle from the same platform, using cell-laden hydrogels protected by biodegradable polymers. But the authors themselves state that further development is aimed at “the production of tissues for human applications and the construction of more complex tissues and solid organs”, which linguistically sounds like an incremental step, but in fact means a leap over the very nature of the problem. Cells are not an inert material: their response depends on the microenvironment that changes minute by minute during the post-printing period. Lee et al. [4] in the FRESH study also demonstrate that cardiomyocytes in 3D-printed ventricular constructs develop synchronized contractions, but only after a period of maturation.

Liu et al. [12], describing the 3D bioprinting of neural tissue for spinal cord repair, themselves state a key obstacle: “bulky printing process, poor cell viability and minimal cell-material interaction”. The term “minimal cell-material interaction” is important because it is not a problem of printing; it is a problem of the biology of the system that printing is trying to recreate. Similarly, Kim et al. [13], in a paper on the integration of neural cells into skeletal muscle constructs, demonstrate that the formation of neuromuscular junctions, long-term survival and orientation of myofibrils depend on interactions that are statistical in nature.

At a more granular level, the variability discussed above resolves into specific mechanical and physiological processes, extrusion–shear-induced membrane deformation as cells transit the nozzle, post-extrusion cytoskeleton recovery kinetics, hypoxia and nutrient gradients during layer-by-layer printing, and stochastic post-deposition maturation driven by cell–cell signaling on time scales of hours to weeks. A thorough mechanistic synthesis of these processes lies outside the scope of the present bibliometric and conceptual review and constitutes a complementary direction; for the argument that follows, the relevant point is that any AI-native architecture (Section 7) must receive signals from these strata rather than averaging over them, which is why dimensions (ii) and (v) of the biological intelligence framework (Section 5) name them explicitly.

This has an important consequence for optimization: even if the system were deterministic in its mechanical parameters, the biological outcome remains distributed. Empirical adjustment that minimizes one numerical indicator (e.g., cell density after 24 h) does not guarantee an optimum functional outcome measured after weeks or months.

### 2.3. Shift from Fabrication Fidelity to Functional Outcome

The third dimension of the problem is conceptual, not technical. For decades, the field has valued its success through fabrication fidelity, i.e., geometric accuracy of the construct in relation to the CAD model, shape fidelity after crosslinking, and mechanical stability. These criteria are important, but they are not enough.

Lee et al. [4], in their work on FRESH heart bioprinting, explicitly set the bar: not only do the constructs reproduce the anatomy of the ventricles with a 20-micron resolution, but cardiac ventricles printed with human cardiomyocytes show synchronized contractions, directional action potential propagation, and wall thickening up to 14% during peak systole. In other words, functional parameters (conductivity, contractility, wall thickening under load) enter the definition of success, not just geometry. This change in benchmark is also essential for the AI layer that we cover in the next section: if the goal is functional outcome prediction, not just printability, then both model inputs and outputs change.

Yan et al. [8] in 2024 bring this shift to its logical extreme. Their approach to printing human neural tissues is validated not through structure, but through functional connectivity, i.e., through cortical–striatal projections, spontaneous synaptic currents, calcium flux, and glutamate transport. A tissue is considered successfully printed if it behaves like tissue. Similarly, Wang et al. [10], in a review on bioprinting for wound healing, emphasize that the success of “smart wound dressings” is no longer a question of structure, but a question of “biostructure and biofunction” which must actively respond to the local biology of the wound. The depth of that local biology is itself substantial: Tottoli et al. [14], in a comprehensive review of skin wound healing and regeneration, detail a multi-phase cascade—hemostasis, inflammation, proliferation, and remodeling—driven by interacting fibroblasts, keratinocytes, and endothelial and immune cells together with a shifting growth-factor profile, and survey the scaffold and 3D bioprinting strategies that must accommodate this dynamic microenvironment rather than reconstruct a static dermal geometry.

This conceptual shift is what makes empirical optimization decidedly insufficient. Functional outcome cannot be optimized directly at the level of bioink or printer settings, but rather emerges from post-printing interactions on time scales far beyond the fabrication process. Optimizing a system that behaves like this requires a tool that models the system itself, not individual parameters. This tool is the subject of the next section.

## 3. Where AI Enters the Bioprinting Pipeline

### 3.1. Organizational Logic of the Section

If the complexity from the previous section is accepted as a real structural problem of the field, the next logical question is not which AI method should be applied, but where in the pipeline AI enters and what function it performs. This review organizes the literature on intersection by function, not by algorithm, and this is a conscious methodological decision, because the same AI method (e.g., convolutional neural network) can serve different goals at different points in the pipeline, while two completely different methods (regression model and Bayesian optimization) can solve the same operational problem (prediction of printability, for example).

The six subsections that follow map the 365 papers at the intersection of bioprinting and AI into six functional domains: bioink design (Section 3.1), process optimization (Section 3.2), real-time monitoring and quality control (Section 3.3), prediction of cell viability and biological outcomes (Section 3.4), scaffold–function mapping (Section 3.5), and clinical translation (Section 3.6). The distribution of works by these categories is highly asymmetric, as shown in Figure 2, where we use that asymmetry as an instrument: it signals which pipeline segments are saturated, and which represent structural gaps in the current state of the field. The functional mapping in this section draws on the full corpus of 365 papers. The algorithm-level analysis in Section 4 (Table 1), by contrast, is restricted to the top-cited subset of 78 articles defined in Section 6.4, for which the methodological detail required to assign a specific algorithm is reliably available.

In addition to this functional dimension (AIF), works can also be mapped by target application (APP): bone, cartilage, skin, vascular, neural, organoid/tumor, liver, cornea, and cardiac. The distribution by applications, shown in Figure S2 of the Supplementary Material, shows the dominance of organoid/tumor models (90 papers) and the clear unavailability of cardiac, hepatic and corneal applications, which is complementary to the asymmetry that we analyze here according to the AIF dimension.

### 3.2. AI for Bioink Design

Ten papers in our analysis treat this domain directly, which makes it, quantitatively speaking, one of the less developed segments of the pipeline. The essential problem that AI solves here is the prediction of the rheological and mechanical properties of bioink from its composition, which classically requires iterative experiments.

The most influential review in this domain, Freeman et al. [15] from 2022 (88 citations, 17.6 citations/year), explicitly positions ML as a tool for “accelerating optimization, enabling real-time error detection, and reducing the number of iterative steps necessary for bioink formulation”. The authors systematically analyze how shear storage, loss moduli, viscosity and shear-thinning properties affect printability. Especially important is that the paper also covers closed-loop AI printing, i.e., an approach where the model not only predicts before printing, but is updated during printing based on the output signal.

Concrete implementations have appeared in the last two years. Sarah et al. [16] develop a model for predicting the viscosity of a hybrid hydrogel bioink based on alginate, gelatin and TEMPO-oxidized cellulose nanofibrils. Using polynomial regression, decision tree and random forest algorithms over 169 rheological measurements, they find that the random forest model achieves R^2^ = 0.99 and MAE = 0.09. A similar approach with ALGEC formulations was provided by Sarah et al. in [17], with the model achieving R^2^ = 0.98 and MAE = 0.12.

Recently, AI-driven 3D bioprinting for regenerative medicine: From bench to bedside [18], 72 citations, 36 citations/year) integrated bioink design into a broader Quality-by-Design (QbD) framework, mapping AI applications through multi-scale and multi-modal sensing, data-driven design, and in-line process control. The logical extreme of the trend is represented by the concept of self-driving bioprinting laboratories [19], where on-demand bioink formulation becomes part of a closed loop that includes autonomous cell cultivation, robotic transplantation and AI-guided printing.

### 3.3. AI for Process Optimization

This category contains 27 papers in our analysis and represents the most technically mature segment of the AI–bioprinting cross-section. Optimization of process parameters (nozzle speed, pressure, temperature, layer thickness, infill density) is a problem that is methodologically well mapped to classic ML approaches.

The conceptual foundation was laid by Yu and Jiang [20] in the paper A Perspective on Using Machine Learning in 3D Bioprinting (201 citations). The strongest operationalization is by Ng et al. [7] in *Adv Mater* 2024 (246 citations, 82 citations/year—the strongest recent impact in the entire dataset), which maps five primary ML applications: quality control, process optimization, design optimization, microstructure analysis, and material formulation. A concrete closed loop for extrusion bioprinting was realized in Bonatti et al. [21] (50 citations), who provided the first demonstration of a total quality-control loop for EBB. A similar approach is applied by Dai et al. [22] on personalized oral grafts, mapping interparametric interactions. Goh et al. [23] extend the methodology to bioelectronic devices.

### 3.4. AI for Real-Time Monitoring and Quality Control

With 38 papers, this category is the largest within Section 3. The difference between Section 3.2 and Section 3.3 is time: in Section 3.2, AI works before/after printing; in Section 3.3, AI works during printing or cultivation, on live data. The most sophisticated approach with a biological outcome is Tebon et al. [24] in Drug screening at single-organoid resolution via bioprinting and interferometry. The authors combine tumor organoid bioprinting with high-speed live-cell interferometry (HSLCI) and ML segmentation, measuring the masses of thousands of organoids in parallel, which moves “monitoring” from the process–technical to biological–functional level.

Al-Kharusi et al. [25] establish a methodological bridge between DoE and ML, explicitly stating that “DoE is only for quantitative data and is not suitable for snapshots and high-dimensional data”; therefore, they propose the integration of DoE and ML into a single system. Tadesse et al. [26] demonstrate surface-enhanced Raman spectroscopy (SERS) for rapid diagnostics, with bioprinting as a platform and ML as an interpretation layer. None of the six most influential works in the category use an isolated signal because each combines at least two modalities, a move toward active signal fusion.

### 3.5. AI for Prediction of Cell Viability and Biological Outcomes

This is the category with the least number of papers in the entire dataset—only three publications for the 10-year period 2015–2026. The asymmetry is dramatic: while clinical translation (Section 3.6) has 154 publications, the most fundamental clinical problem accounts for less than 2% of that volume. This is not a casual observation, but an essential structural finding of this review.

The most significant work in the domain is Mohammadrezaei et al. [27] in Cell viability prediction and optimization in extrusion-based bioprinting via neural network-based Bayesian optimization models (*Biofabrication* 2024, 31 citations). The trained regression neural model achieves R^2^ = 0.71, and the classification model achieves 86% accuracy. Methodologically interesting is that the paper introduces Bayesian optimization combined with a neural network regressor to maximize viability. The performance of the model itself reflects the limit of the current understanding of the system; i.e., prediction of mechanical properties of bioink achieves R^2^ = 0.99, while prediction of biological outcome achieves only 0.71. It is diagnostic information. Iglesias et al. [28] (*J Craniofacial Surg* 2026) from a surgical perspective set the framework for what remains to be done.

The consequence is that without biological feedback, the closed-loop systems described in Section 3.2 and Section 3.3 remain closed only in the technical sense—sophisticated but incomplete automation.

### 3.6. AI for Scaffold–Function Mapping

With 13 papers, this category is nominally medium in size, but a close reading reveals that only one paper treats AI as a central methodology, while the others mention AI as a future perspective. The domain is in the conceptual phase. A specific reference point is Stewart et al. [29] in Machine intelligence for nerve conduit design and production; they explicitly map machine intelligence, high-dimensional image analysis, and computational scaffold design as three convergent forces for the design of nerve guidance conduits.

There are conceptual signals in recent review papers—Branco et al. [30] (GBM models), Li et al. [31], Gebeshuber et al. [32] (biomimetic scaffolds), and Liu et al. [33] (alginate-based) synchronously mention AI as the “next step” without concrete implementations. This is a signal that the field anticipates a methodological shift but has not yet realized it. The scaffold–function domain is waiting for Section 3.4 to be developed.

In the related domain of electrospinning, Virijević et al. [34] in 2024 demonstrate an AI-driven workflow where the ANN model guides scaffold selection through the entire validation chain: from cytotoxicity, through CAM neoangiogenesis, to in vivo healing on a rat burn model. Although the work is not from bioprinting, the methodological logic, where AI serves as a layer connecting material and biological outcome, illustrates the target state for scaffold–function mapping.

### 3.7. AI for Clinical Translation

With 154 papers, this is the largest category in the entire dataset, dominating the other five combined. Its size signals that the field has gone “from the exit to the entrance”: clinical application is the dominant research topic, while the basic scientific layers are lagging behind. However, “translation” is not monolithic, but consists of three recognizable currents: (i) personalized implant production, (ii) clinically specific applications, and (iii) AI-driven systemic frameworks for QbD-based development.

The strongest operationalization is AI-driven 3D bioprinting [19], which appears for the third time and represents an integrative framework that connects all downstream segments. Nagarajan et al. [35] define the target image, patient-specific anatomy, pathology and biomechanical properties. Clinically specific applications include: Inkjet Printing of Pharmaceuticals [36] with pharma-inks, Sun et al. [37] with bone defects, and Wang et al. [38] with organoid models for cancer immunotherapy. Nahak et al. [39] set up an OoC ecosystem; Ray RR [40] on periodontitis is a peripheral paper that illustrates the result of a broad filter.

Synthesis point of the category: 154 papers do not represent mature clinical translation, but rather research commitment to clinical translation that is often not supported by mature infrastructure in downstream segments (viability, scaffold–function). The volume signal here should be read with care: a large number of papers in a category measures research focus, not clinical evidence. The 154 publications in Section 3.6 are dominated by review articles, conceptual frameworks, and demonstration studies. The fraction that constitutes original experimental work meeting standards of clinical-translational evidence (controlled comparison, defined endpoints, regulatory-grade methodology) is substantially smaller, and this manuscript does not attempt to break it down at that level; a more granular classification of clinical evidence strength within Section 3.6 is a natural follow-up to the present bibliometric mapping. In other words, the field is moving towards translation before it has fully understood what is required to support it, and the visible publication volume is a leading indicator of intent rather than a lagging indicator of accomplishment.

## 4. AI Methods × Functions: Mapping to Concrete Implementations

### 4.1. Why Map a Method to a Function?

The standard way of organizing the AI literature is by algorithm: neural networks in one chapter, random forest in another, and reinforcement learning in the third. This approach has educational value but hides operational information: in a real implementation, the choice of algorithm arises from the nature of the problem, not the other way around. A convolutional neural network (CNN) is an expected choice for a problem involving real-time images; Bayesian optimization occurs naturally where measurement is expensive and the parameter space is high-dimensional; and random forest dominates where the inputs are tabular and the amount of data is modest.

### 4.2. Mapping of AI Methods to Functional Domains

Table 1 summarizes this mapping for the top-cited subset of 78 articles (defined in Section 6.4), a deliberately narrower base than the 365-paper corpus mapped functionally in Section 3. Algorithm specification is consistently reported only in highly cited work, so restricting the table to this subset avoids over-interpreting the sparse methodological reporting in the long tail of the corpus. Fields marked with “—” indicate combinations where we did not identify a specific implementation in our dataset.

**Table 1:** AI methods × functions in 3D bioprinting (transposed orientation: categories are rows; methods are columns). The table reflects only the top-cited subset of the corpus (see Section 6.4), not the full 365-paper dataset; fields with “—” indicate combinations for which no explicit algorithm was documented in that subset, not necessarily an absence across the entire corpus. Papers that operationalize more than one functional role are listed in every applicable row rather than being forced into a single category: for example, the closed-loop system of Bonatti et al. [21] uses a single convolutional neural network to both optimize printing parameters (Section 3.2) and monitor the print online (Section 3.3), and therefore appears in both rows under CNN. This cross-domain placement is deliberate and mirrors the multi-category classification documented in Table S3 of the Supplementary Material. The “Generic ML” column lists studies applying or discussing machine learning for the corresponding task without specifying a named algorithm or architecture.

AIF Category	Generic ML	Polynomial/Linear	Random Forest	CNN	NN (General)	Bayes Opt.	ANN	ML + DoE	ML + HSLCI
3.1 Bioink	[15,18,19]	[16,17]	[16]	—	—	—	—	—	—
3.2 Process	[7,18,20,22,23]	—	—	[21]	—	—	—	—	—
3.3 Monitoring	[7,20,26]	—	—	[21,24]	—	—	—	[25]	[24]
3.4 Viability	[28]	—	—	—	[27]	[27]	—	—	—
3.5 Scaffold	[30,31,32,33]	—	—	—	—	—	[29]	—	—
3.6 Translation	[18,35,36,37,38,39]	—	—	—	—	—	[40]	—	—

Note: Generic ML/AI without algorithm specification (the largest category in each column) is not shown due to space constraints. This includes: 3.1 [15,18,19]; 3.2 [7,18,20,22,23]; 3.3 [7,20,26]; 3.4 [28]; 3.5 [29,30,31,32,33]; and 3.6 [18,35,36,37,38,39].

### 4.3. What Does the Table Reveal?

First, the table reveals the concentration of specification. Categories 3.1 (bioink) and 3.4 (viability) are the only two where the top-cited papers explicitly specify the algorithm. These are not random but are domains where the experimental dataset is limited and where it is justified to document the choice of algorithm as part of the methodological argument.

Second, the table reveals CNN distribution. Convolutional neural networks appear explicitly only in categories 3.2 and 3.3 with direct image processing. The absence of a CNN specification in 3.5 (scaffold) is interesting because the geometric design of a scaffold is theoretically an ideal case for 3D-CNN architectures, but in the top-cited papers, that space remains unfilled.

Third, the table reveals Bayesian optimization as a silent differentiator. Mohammadrezaei et al. [27] (3.4) introduce Bayesian optimization as a methodological response to the specificity of the viability problem, i.e., to the high costs of experiments, and then to a limited dataset. The fact that this paper is the reference in the category with the fewest publications is a signal that the methodological choice follows the nature of the problem.

Fourth, the table reveals empty fields as a research map. Fields with “—” are explicit research blanks. None of the top-cited papers in Section 3.1 use CNNs, although images of bioink structure and fluorescence during crosslinking are realistic inputs; no work in Section 3.5 uses Bayesian optimization for topology design. These places are the natural next point for the AI-native generation of bioprinting research.

Finally, the table provides a signal about the maturation of the field. In the classic AM literature, generative adversarial networks (GANs), reinforcement learning, and transformer models have already been documented. None of these three approaches appear explicitly in the top-cited part of our bioprinting literature. The gap between the AM and bioprinting literature at the methodological level is measurable.

## 5. From Printing Accuracy to Biological Intelligence

### 5.1. The Current Definition of Success and Its Limitations

3D bioprinting as a field has historically developed its evaluation system from additive manufacturing (AM) in a broader sense. The criteria that have been transferred—geometric accuracy, shape fidelity, mechanical stability, printability, and dimensional accuracy—are valid and useful. But a careful reading of concrete demonstrations from recent works reveals that these criteria, by themselves, are not sufficient.

Lee et al. [4], in their FRESH paper on human heart printing, demonstrate 20-micron resolution and structural reproduction of the anatomy, but validation lies in the fact that cardiomyocytes show synchronized contractions, propagation of action potentials, and 14% wall thickening during peak systole. Yan et al. [8] in 2024 validate print through cortical–striatal projections, spontaneous synaptic currents, and calcium flux, where geometric accuracy ceases to be a relevant metric; the result is whether the tissue functions as a tissue.

This pattern is repeated throughout recent AI–bioprinting work. Tebon et al. [24] measured the masses of individual organoids over time. Mohammadrezaei et al. [27] model viability, not geometry. Wang et al. [10] emphasize biostructure and biofunction. In practice, the field has already moved from printing accuracy metrics, but this is not always reflected in the language in which the results are reported.

### 5.2. What Is Biological Intelligence—A Definition of the Term

**Figure 3:**
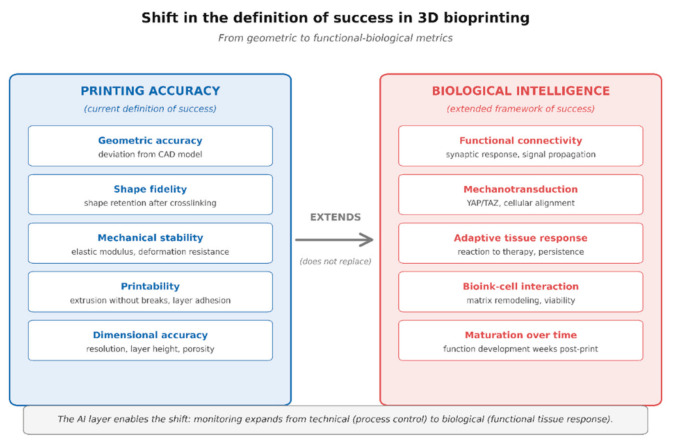
A shift in the definition of 3D bioprinting success. (**Left**): traditional printing accuracy framework (5 geometric/mechanical-type metrics). (**Right**): proposed extended framework of biological intelligence (5 dimensions of functional–biological metrics). Biological intelligence extends, not replaces, the printing accuracy framework.

We propose the term biological intelligence as an extended framework for evaluating 3D bioprinting systems. The term is not new in its basic form—it has been used in synthetic biology and other fields—but in the context of bioprinting, it acquires a specific operational content. In our definition, it includes five complementary dimensions (Figure 3):

(i) Functional connectivity—the ability of the construct to establish and maintain signaling networks appropriate to the tissue type.
(ii) Mechanotransduction—the capacity of cells to respond to mechanical signals (matrix stiffness, loading), including YAP/TAZ signaling and cytoskeletal reorganization.
(iii) Adaptive tissue response—the ability of the construct to change its properties in response to interventions over longer periods of time.
(iv) Bioink–cell interaction—dynamic interaction between matrix material and cells, including matrix remodeling and progressive replacement of synthetic material by biological ECM.
(v) Temporal maturation—the system’s capacity to mature to a functional state in the weeks or months following printing.

To clarify the relationship between this proposal and established paradigms, the five dimensions map onto, rather than compete with, three existing frameworks. Functional tissue engineering, which has long measured success by in-service behavior rather than structural facsimile, is operationally captured in dimensions (i) functional connectivity and (ii) mechanotransduction. The structure–function axis of classical biomaterials research runs through (iv) bioink–cell interaction and (v) temporal maturation, where structural choices influence outcomes only through the named biological dynamics. 4D bioprinting [9] adds a post-fabrication time axis, which is translated into AI-trainable quantities through (iii) adaptive tissue response and (v) temporal maturation. Biological intelligence is therefore a convergence of these frameworks, anchored in a vocabulary through which closed-loop AI-native systems can directly act.

The key point is that biological intelligence does not replace printing accuracy but rather extends it. Geometric accuracy remains a prerequisite; biological intelligence becomes the goal. A construct that is not geometrically accurate cannot be biologically intelligent, but geometric accuracy alone does not constitute a functional tissue.

### 5.3. Operational Implications of the Shift

First, the shift redefines what AI predicts. In the classic formulation, AI predicts process output; in the expanded framework, it predicts biological outcome (viability, functionality, therapeutic response). This difference changes the objective function of the model, the nature of the training data, and the validation metrics.

Second, the shift redefines what AI must measure. Classic equipment (camera, load cell, rheometer) measures process parameters. Biological–functional metrics require a new class of sensors, such as calcium recording, patch clamp, organoid mass (as in Tebon et al. [24]), and electrophysiological monitoring.

Third, the shift redefines what constitutes a closed-loop system. Bonatti et al. [21] demonstrated a technically closed loop, but it is a technical closed loop, where the system knows nothing about the biological outcome. A true biological closed loop, which Ref. [18] predicts through the QbD framework and which Self-driving bioprinting laboratories [19] sets as a horizon, seeks feedback not from a camera, but from a biological sensor.

Fourth, the shift redefines research priorities. If the goal is biological intelligence, then 3.4 (viability, 3 publications) and 3.5 (scaffold–function, 13 publications) are the most critical areas for development, not 3.6 (translation, 154 publications). The current distribution reflects a field that has moved “from the exit to the entrance”. However, in the biological intelligence framework, the priorities are reversed.

## 6. Current Limitations of AI–Bioprinting Integration

Although the previous sections show that the field has made significant progress, there are several classes of limitations that prevent the realization of the biological intelligence framework in practice. This section systematizes the constraints into four groups.

### 6.1. Data Restrictions

The dataset size is small, especially for biological outputs. Mohammadrezaei et al. [27] explicitly combine their own measurements with data from the literature, which is a signal that no laboratory alone has enough data. Freeman et al. [15] in their review could not identify work with more than ~200 data points. Classical ML implementations operate on two orders of magnitude less data than in other domains.

*Publication bias* is a systematic problem. Negative results are not reported, which means that ML models are trained on an incomplete distribution. Protocol heterogeneity further reduces the usability of published data. Bonatti et al. [21] explicitly emphasize the goal of “standardization of results across multiple laboratories”, which is a signal that standardization has not been achieved.

### 6.2. Model Limitations

Overfitting and limited transferability are the main risks of small datasets. A model achieving R^2^ = 0.99 on 169 measurements in Sarah et al. [16] does not necessarily generalize to a bioink composition outside the training area, nor to a printer of another manufacturer. The lack of inter-laboratory validation in the top-cited papers is striking.

Pseudo-validation is a more subtle problem because “AI model validation” often boils down to internal cross-validation on the same dataset, not independent validation. The black box problem is relevant in the clinical context, because when Mohammadrezaei et al. [27] achieve 86% classification accuracy, the regulator (FDA, EMA) can ask for an explanation why, and the neural network cannot directly answer. Concrete implementations of interpretable AI in bioprinting are rare.

### 6.3. Evaluation Limitations

Standardized metrics for biological outcomes do not exist. Geometric accuracy is measured in micrometers with CT/MRI comparison. But how is the “functional connectivity” of a neural construct measured? Yan et al. [8] measure the number of synaptic currents per cell; another paper could measure the propagation speed, and a third could measure the synchronization of oscillations. Without common metrics, AI models from different labs cannot validate each other. The same applies to the adaptive tissue response: Tebon et al. [24] measure organoid masses, but what constitutes a “successful” answer depends on the context.

### 6.4. Limitations of This Review

Three specific limitations of our approach must be explicitly stated.

Top-cited sampling (top 5 all-time + top 5 recent_impact per query, 78 unique papers; the complete reading-list construction methodology is described in Section S1.4 of the Supplementary Material) is biased against fresh papers that have not yet accumulated citations. No explicit [Language] filter was applied at the query level; non-English work is therefore under-represented to the extent that PubMed indexing under-represents it, particularly research published in regional databases in China, South Korea, and Japan. The temporal latency of citations favors older material; the cit/yr metric balances out partially, but not perfectly. These limits do not disqualify the conclusions, but they do qualify them. The trends (10.4× growth, 136× intercept, asymmetry by AIF categories) are robust because the signals are at the field level. A specific concern—that the 154 vs. 3 asymmetry might reflect citation bias rather than a real research distribution—admits a direct check. In the full 365-paper corpus, the translation-to-viability ratio is ~51:1; in the top-cited subset of 78 papers (Section 6.4), the same categories appear with seven and two publications, respectively, a ratio of ~3.5:1. If citation accumulation were inflating translation over viability, the most-cited subset would widen the gap, not narrow it. The observed direction is the opposite, so the headline asymmetry reads as a research-focus signal rather than a citation-skewing artifact; viability remains the substantively underdeveloped category regardless of framing.

## 7. AI-Native Bioprinting: The Next Phase of the Field

### 7.1. AI-Augmented vs. AI-Native—A Terminological Difference That Changes Everything

The current field, as we described it in Section 3, is most accurately described as AI-augmented bioprinting, which is a traditional system (bioink, printer, cell culture) with AI components added at certain points. AI in this mode is a tool to improve the existing approach, not a structural element.

AI-native bioprinting represents a qualitatively different concept: a system in which the AI layer is an architectural prerequisite. The difference is analogous to that between a car with a computer to control the injectors and an autonomous vehicle, where in the former the AI optimizes the existing subsystem, and in the latter the AI defines what the system can do in general.

Specifically, (i) AI is no longer a post hoc analytical layer, but a real-time operational component; (ii) the system learns continuously from each print; (iii) biological signals are equal to process signals in the control loop; (iv) the human operator becomes the supervisor. Self-driving bioprinting laboratories [19] is currently the clearest articulation of this concept, where the authors explicitly use the term “*fully integrated, autonomous, closed-loop system capable of designing, fabricating, maturing, and assessing living tissue constructs*”.

### 7.2. Four Components of an AI-Native System

(1) Multi-modal sensing layer. This denotes not one camera, but a continuous, parallel flow of signals from different modalities: visual, mechanical, and biological (calcium recording, electrophysiology, secretome analysis). The AI layer works on their fusion. As a prospective design direction—and one not yet documented in the bioprinting + AI corpus we surveyed—transformer-like architectures emerge as natural candidates for this multi-modal fusion task; we present this as a research-direction signal rather than an empirical claim derived from the dataset.
(2) Continuous learning layer. Classical ML is trained on a fixed dataset and does not learn more. The AI-native system updates its models from each new print run, which requires both technical infrastructure (online learning, experience replay) and organizational infrastructure (model versioning). The authors of [16] explicitly mention in-line process control as a component of the QbD framework.
(3) Biological closed loop. The difference between a technically closed loop [21] and a biologically closed loop is essential, because biological feedback requires sensors that work on the time scales of biology (hours, days, weeks), not seconds. The AI layer that processes such a signal is methodologically closer to Bayesian optimization [27] than to CNN classification [21].
(4) Interpretable and human-supervisable layer. AI-native does not mean “automated without human insight”. A human supervisor remains necessary for regulatory, ethical, and research reasons. It requires explainable AI methods, interactive dashboards with uncertainty predictions, and a versioning system for reproducibility.

#### An Illustrative AI-Native Workflow

To make the four components concrete, consider an AI-native workflow for a vascularized cardiac patch, a use-case that exercises every layer. This is presented as a worked example, not a system already realized in our corpus; its constituent technologies all appear in the works cited below, but none of the 365 papers in our intersection integrates them as a single architecturally designed pipeline. This gap is precisely what defines the current state as AI-augmented rather than AI-native.

Sensors: a coaxial extrusion print head equipped with (i) high-speed visual imaging for filament geometry, extending the closed-loop CNN architecture of Bonatti et al. [21] from a single signal to a fused multi-modal input; (ii) impedance spectroscopy at the nozzle for in-process bioink rheology, providing a millisecond-scale signal complementary to the visual one; and (iii) post-deposition holographic live-cell interferometry, analogous to the high-speed live-cell interferometry of Tebon et al. [24] but operating during the print rather than as a post hoc readout, providing early biological signal—cell density and scattering profile correlated with viability—i.e., within minutes of deposition.

Learning methods: A Bayesian neural network ensemble with experience replay, conceptually extending the Bayesian-optimization-plus-neural-regressor architecture of Mohammadrezaei et al. [27] from offline training to online updating. The model maintains a posterior over the mapping (bioink composition + process parameters + cell line) → (printability, immediate viability, day-7 functional connectivity). Each completed print produces a labeled tuple that updates the posterior; model weights are versioned and rollback is automatic if cross-validated performance on a held-out reference panel degrades. Generative components (a diffusion model in the manner of category 3.5 in Table 2) propose new bioink-and-geometry candidates outside the current training envelope.

Biological feedback: Operating on three time scales rather than one. Seconds: Visual and impedance signals close the loop to printer actuators (nozzle pressure, translation speed). Hours: Calcium imaging in cardiac myocytes adjusts the in-incubator maturation protocol (electrical pacing frequency, mechanical stretch amplitude). Days: Electrophysiology and contractility readings, in the spirit of the functional validation paradigm of Yan et al. [8] for neural tissue, feed back not to the current print but to the next batch—adjusting the bioink formulation and the maturation timeline for the subsequent construct. This last loop is what “native” means architecturally: the system is designed to learn between prints, not within a single print only, and the slow biological signal is the dominant signal, not an afterthought to the fast process signal.

Human supervision: An interactive dashboard surfaces per-print uncertainty bands, between-sensor agreement diagnostics, and model-version metadata. A human reviewer signs off on any predicted change to the bioink formulation or maturation protocol that exceeds predefined safety thresholds; below those thresholds, the system updates autonomously and logs the decision for retrospective audit. This is consistent with the regulatory framing of Quality-by-Design pipelines such as those reviewed in [18], where automation and human accountability coexist by design rather than in tension.

Where on the spectrum: The four components above define the design axes along which any concrete system sits. A workflow that implements only sensing fusion (i) and a fixed-weight predictor (the static part of (ii)) is still AI-augmented—the AI improves the existing pipeline but is not its precondition. Adding online learning makes the system partially AI-native on the learning axis. Adding the slow biological loop in (iii) extends nativeness to the feedback axis. Only when all four axes are co-designed—including (iv), where explainability is treated as a first-order requirement rather than a post hoc add-on for the regulators—does the workflow become AI-native in the full architectural sense. Most current systems in our corpus operate on one or two of these axes natively and on the others in augmented mode; the transition path described in Table 2 is the gradual extension of nativeness along all four axes simultaneously rather than the addition of one more AI tool to an existing pipeline.

### 7.3. Current vs. AI-Native State Across AIF Categories

The transition is not smooth. Categories 3.1 and 3.2 have a shorter path; existing systems can be incrementally expanded. Categories 3.4, 3.5 and especially 3.6 require fundamentally new components. This is consistent with the finding of Section 5 that research priorities in the perspective of AI-native approaches differ substantially from the current distribution of publications.

**Table 2:** Mapping of current state (AI-augmented) and target state (AI-native) across six functional categories defined in Section 3.

Category	Current State (AI-Augmented)	AI-Native State
3.1 Bioink	Prediction of viscosity from composition [16,17]; QbD framework [18]	On-demand closed-loop formulation of mechanical and biological tests [19]
3.2 Process	Optimization of parameters before printing [7,20,22]; quality-control loop [21]	Adaptive real-time system that adjusts parameters based on online sensing
3.3 Monitoring	Post hoc analysis and online CV [21,24]; ML + DoE [25]	Multi-modal sensor fusion with parallel visual, mechanical, and biological signals
3.4 Viability	NN + Bayesian optimization on limited datasets [27]	Continual learning from every press; biological signals as primary input
3.5 Scaffold	Conceptual signals; machine intelligence for NGCs [29]	Generative models (GAN, diffusion) for topological design guided by functional outcomes
3.6 Translation	Personalized production [35,36,37]; QbD framework [18]	Closed-loop clinical valorization with biological feedback

### 7.4. What Exactly Does the Field Need to Do?

(a) Data openness. Without shared datasets, ML models remain locked in labs. The field needs a standardized format for bioink composition + process parameters + biological outcome.
(b) Consensus metrics for biological outcomes. Formation of working groups (similar to ISO/ASTM committees) to define metrics for functional connectivity, mechanotransduction, and adaptive response with quantitative protocols is needed.
(c) Inter-laboratory validation as a rule. Models working in a single laboratory should not be published as “validated” until they have undergone independent replication.
(d) Regulatory engagement sooner, not later. Cooperation with regulators (FDA, EMA) in the design phase of AI systems is more effective than retroactive compliance.
(e) Education and transdisciplinary personnel. AI-native bioprinting is looking for a new generation of researchers who are simultaneously biologists, engineers, and connoisseurs of AI methodology. It requires long-term investments in educational infrastructure.

## 8. Conclusions

3D bioprinting has matured over the last decade to the point where empirical optimization no longer scales with system complexity, while the intersection with artificial intelligence has grown 136-fold: from one publication in 2015 to 136 in 2025. This work has shown that AI in bioprinting is no longer an auxiliary tool, but an operational layer of intelligence that is reshaping how the field is conceptualized, how success is measured, and how research priorities are organized.

A systematic analysis of 365 papers across six functional categories reveals a distinct asymmetry: while clinical translation dominates with 154 publications, cell viability prediction counts only three. This disparity shows that the field has gone “from the output to the input”, which in the short term allows for rapid clinical demonstrations, but in the long term feeds the basic science gaps that each subsequent generation of systems will encounter.

We have argued that overcoming these gaps requires two complementary shifts. The first is conceptual, going from printing accuracy metrics to the framework of biological intelligence, which defines the success of the system not through geometry, but through functional connectivity, mechanotransduction, and adaptive tissue response. The second is architectural, where it goes from AI-augmented systems (AI as an addition to the existing pipeline) to AI-native systems in which AI is a structural prerequisite, with continuous learning, multi-modal sensing, and biologically closed loops.

The roadmaps that follow from this perspective are concrete: open and standardized datasets, consensus metrics for biological outcomes, inter-laboratory validation as a rule, early collaboration with regulatory bodies, and a transdisciplinary educational infrastructure. The field is mature enough to systematically address them. The next decade of bioprinting will not be defined by how fast we can print, but by how intelligently we print.

## Data Availability

No new data were created or analyzed in this study. Data sharing is not applicable to this article.

## Supplementary Materials

The following supporting information can be downloaded at https://www.mdpi.com/article/10.3390/jfb17070319/s1. Table S1: Annual publication counts for the three populations analyzed in this review—all 3D bioprinting literature (BIO), all AI literature (AIM), and their intersection (BIO × AIM)—for the period 2015–2026, retrieved via the NCBI Entrez API (search executed March–April 2026). Fold-changes are computed over the complete-year window 2015–2025; the year 2026 is partial (data through April 2026) and is reported for completeness but excluded from fold-change and regression calculations; Table S2: Overview of all queries and their results; Table S3: Papers appearing in multiple AIF categories; Table S4. Pair-wise overlap of AIF sub-axes (unique PMIDs shared). Diagonal entries give the per-query unique total; off-diagonal entries give the number of PMIDs shared between two sub-axes. Snapshot: 29 May 2026; Figure S1: Growth of publications in a linear scale; Figure S2: Distribution of publications by target applications (APP); Figure S3: Distribution of publications by AI function (AIF).
