# Supplementary material for: The Next Phase of 3D Bioprinting: AI-Native Systems—A Narrative Review
Source: J Funct Biomater. 2026 Jul 3;17(7):319. doi: 10.3390/jfb17070319 (PMC13412512; doi:10.3390/jfb17070319)
Supplement: Supplementary file 1 [file jfb-17-00319-s001.zip › jfb-4333999-supplementary.pdf]

Supplementary Materials

Next Phase is AI-Native 3D Bioprinting

Supplementary material contains a detailed description of the systematic literature search methodology, additional figures, and a table of references with quantitative results that are not included in the main text.

Authors: Nebojša Zdravković, Mateja Zdravković, Marko Živanović
Univerzitet u Kragujevcu

S1. Literature search methodology

A systematic literature search was conducted during March and April 2026, using publicly available databases and open API services. The goal was to cover the period 2015–2026, with a focus on the intersection of 3D bioprinting and artificial intelligence. To ensure reproducibility, all methodology is structured through four steps described below.

S1.1 The structure of the four search axes

The search is organized through four key word axes, each with its own set of terms:

• **Axes BIO** — terms describing 3D bioprinting technology (15 terms + MeSH "Bioprinting"). Example: "3D bioprinting", "extrusion bioprinting", "bioink", "biofabrication".

• **Axes AIF** — the AI function in the pipeline, divided into 6 sub-categories: bioink design, process optimization, real-time monitoring, viability prediction, scaffold/function mapping, and clinical translation. Each pod has 7-12 specific terms.

• **Axes AIM** — artificial intelligence method, divided into 7 categories: general AI, machine learning, deep learning, computer vision, generative models, reinforcement/Bayesian, and digital twin. The generic term includes *artificial intelligence, machine learning, deep learning, neural network, predictive model* and corresponding MeSH terms.

• **Axes APP** — target application/tissue, divided into 9 categories: bone, cartilage, skin, vascular, neural, organoid/tumor, liver, cornea, cardiac. Each APP category has 4-12 specific terms.

The complete YAML configuration file with all axis terms is available in the supplementary repository (see S1.6).

S1.2 PubMed query construction

The search was conducted through PubMed (NCBI Entrez API). Each query is constructed by a combination of axes, with filters:

• Time range: 2015–2026 (*Date - Publication*)

• Publication type: Review OR Journal Article

• Language: no explicit [Language] filter applied (corpus is English-dominant de facto due to PubMed's indexing profile)

A total of **18 queries** were conducted through the Entrez API:

• 3 baseline queries: total bioprinting literature (BIO), total AI literature (AIM), and their direct intersection (BIO × AIM).

• 6 AIF queries: intersection of BIO × AIM × AIF for each of the six subfunctions.

• 9 APP queries: intersection of BIO × AIM × APP for each of the nine applications.

Each query returned a list of PubMed IDs (PMIDs), without abstract extraction in the first step. The consolidated result of the BIO × AIM intersection yielded a total of 365 unique PMIDs for the period 2015–2026, with the annual distribution shown in Table S1.

Table S1:

Annual publication counts for the three populations analyzed in this review — all 3D bioprinting literature (BIO), all AI literature (AIM), and their intersection (BIO × AIM) — for the period 2015–2026, retrieved via the NCBI Entrez API (search executed March–April 2026). Fold-changes are computed over the complete-year window 2015–2025; the year 2026 is partial (data through April 2026) and is reported for completeness but excluded from fold-change and regression calculations.

Year	ALL bioprinting (BIO)	ALL AI (AIM)	Intersection (BIO × AIM)
2015	158	9,438	1
2016	327	10,388	2
2017	433	12,573	3
2018	542	17,076	4
2019	683	23,962	8
2020	906	33,647	20
2021	1,101	47,266	17
2022	1,202	59,811	32
2023	1,255	63,568	39
2024	1,302	79,370	43
2025	1,648	109,673	136
2026*	663	42,576	60
**Fold-change (2015–2025)**	**10.4×**	**11.6×**	**136×**

S1.3 Citation extraction through OpenAlex

For each PMID identified in the previous step, the citation count and metadata were extracted through the OpenAlex API (https://api.openalex.org/works), using polite-pool mode with email identification. OpenAlex was chosen for three reasons: (i) completely open and free access; (ii) coverage of biomedical literature comparable to Web of Science and Scopus; (iii) a detailed metadata set, including authors, year, journal, DOI, and citation reference numbers with timestamps.

The following were extracted from each item: total number of citations (cited_by_count), year of first indexation, journal, list of authors, DOI. Additional metrics were calculated from this data:

```
cit_per_year = cited_by_count / max(1, current_year - publication_year)
```

which was used as a normalized measure of impact that reduces bias against recent work.

S1.4 Construction of a reading list

They were selected from each of the 18 queries:

- Top 5 works by total citations (alltime impact)
- Top 5 works by cit_per_year (recent impact)

After deduplication (one paper can be top in several axes at the same time), the consolidated reading list included **184 unique papers**. For 78 of these, the full paper is available through the NCBI eFetch API and has been extracted for detailed content analysis in Section 3 of the main paper.

S1.5 Mapping of papers to functional categories

Each paper from the reading list is mapped to six functional AIF categories (Sections 3.1–3.6 of the main paper) based on:

- Title of paper
- Abstract
- The term structure of the query from which the paper was identified

The mapping was controlled through a review of the original papers. Papers that belonged to borderline cases (eg papers that could be classified as both 3.2 and 3.3) were kept in the dominant category according to the central thesis of the paper, and secondary classified according to the method (Table 1 of the main paper).

S1.6 Reproducibility

All used configuration file (YAML with search terms) and helper scripts (Python 3, using only open APIs: NCBI Entrez, OpenAlex, Crossref) are available in the public GitHub repository. A stable version with a DOI number will be deposited on the Zenodo platform before publication of the work.

GitHub repository: *https://github.com/zivanovicmkg/bioprinting-ai-mining-framework*

Zenodo DOI: *10.5281/zenodo.19885393*

The repository contains:

- config/search_terms.yaml — all 18 queries with all axis terms
- scripts/literature_search.py — Entrez query execution
- scripts/top_cited.py — OpenAlex citation extraction
- scripts/build_master_unique.py — deduplication and master list construction

- scripts/fetch_abstracts.py — eFetch abstract extraction
- scripts/field_analysis.py — generating numerical summaries and figures

All scripts use only free and open APIs. The total execution time of the complete pipeline, from the initial search to the final figures, is less than 30 minutes. This allows any researcher to reproduce the analysis or extend it to new research axes.

S2. Additional figures

Three additional figures complement the main text figures and provide a complementary view of the data. Figure S1 provides a linear version of publication growth (Figure 1 in the main paper is a log-scale). Figures S2 and S3 show the distributions by application (APP) and function (AIF) as bar charts, supplementing the heatmap from Figure 2 of the main paper.

Figure S1 — Growth of publications in a linear scale

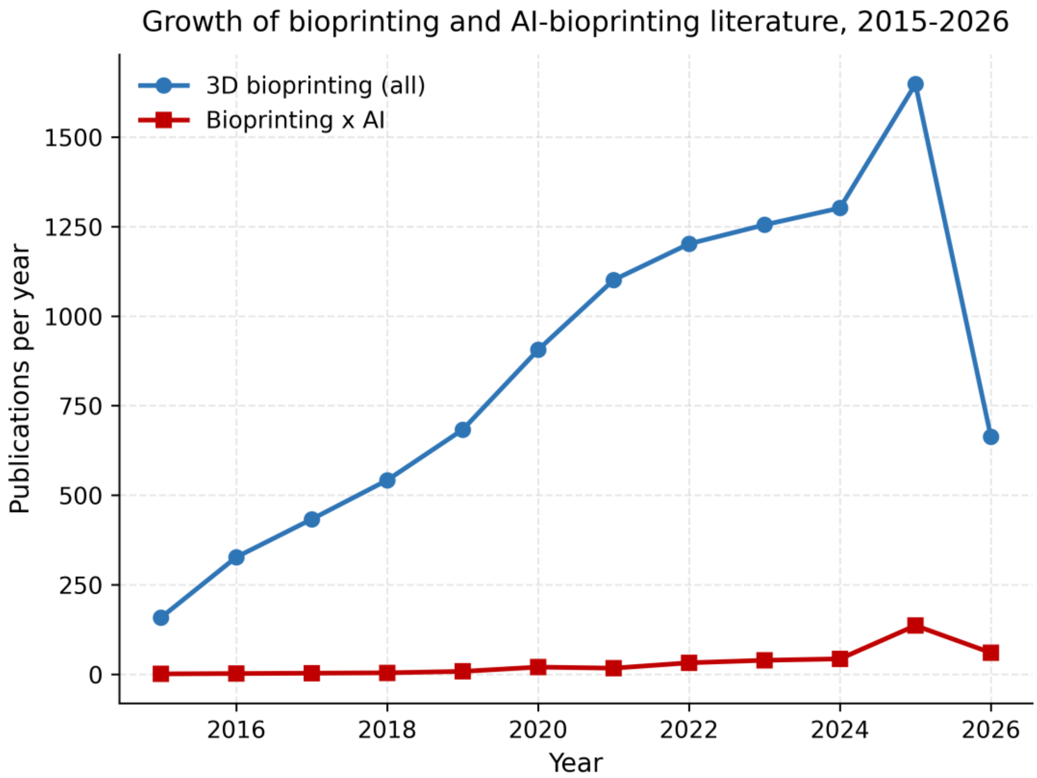

Figure S1. Linear representation of the annual production of publications in 3D bioprinting (blue) and cross section with AI literature (red) for the period 2015–2026. The total bioprinting literature grows from 158 publications in 2015 to 1,648 in 2025. The intersection with AI grows from 1 publication in 2015 to 136 in 2025. The acceleration in the 2024–2025 period (43 → 136 publications, multiplier 3.16×) is also visible in a linear scale. The year 2026 is shown with incomplete data (until April 2026).

Figure S2 — Distribution of publications by target applications (APP)

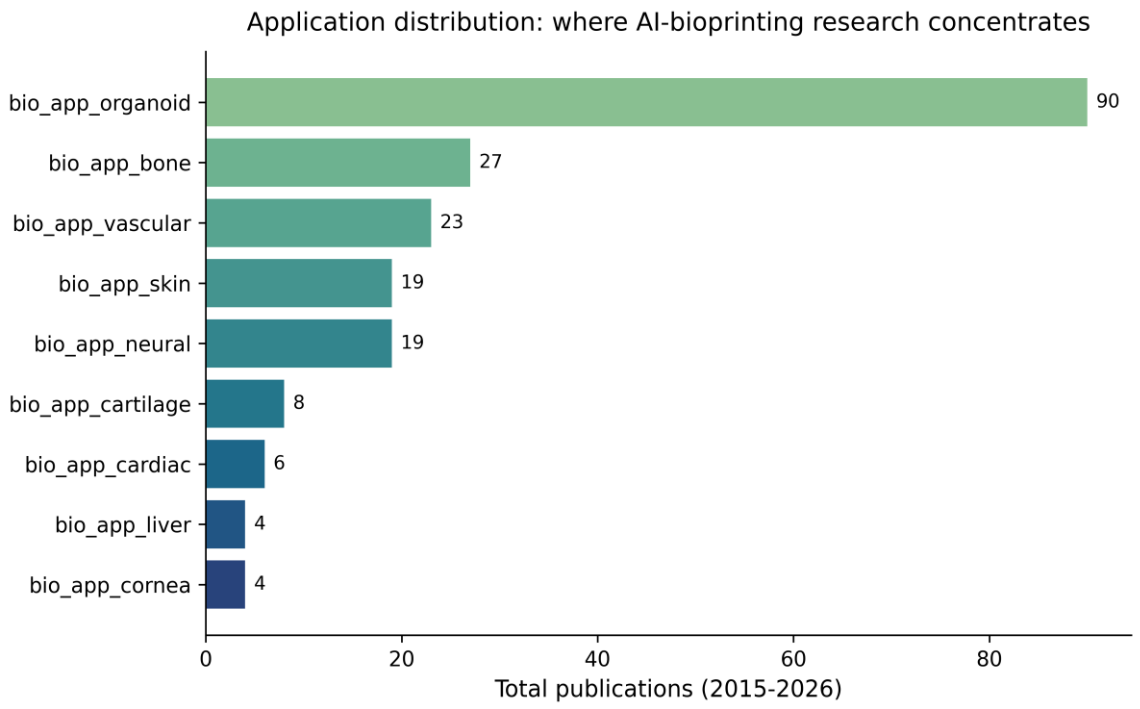

Figure S2. Distribution of publications at the intersection of 3D bioprinting and AI by nine target applications (APP), period 2015–2026. Organoids and tumor models dominate with 90 publications, reflecting the relevance of this domain to clinical translation (3.6 in the main paper) and the availability of AI tools for high-throughput screening (see Spinelli et al. [24]). Bone (27), vascular (23), skin (19), and neural (19) make up the second layer of representation. Cardiac, liver and corneal applications are the least represented (4-6 papers each), although they are the most clinically complex and the most ambitious in research. This distribution supplements the AIF analysis from the main text.

Figure S3 — Distribution of publications by AI function (AIF)

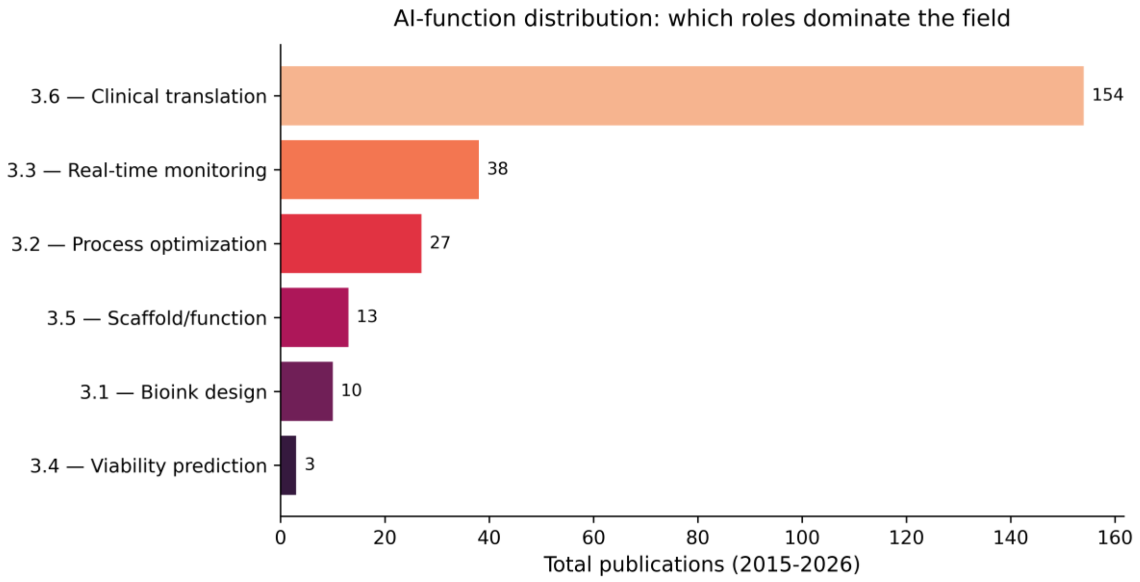

Figure S3. Distribution of publications at the intersection of 3D bioprinting and AI by six functional categories (AIF), period 2015–2026. Clinical translation (3.6) dominates with 154 publications, while cell viability prediction (3.4) counts only 3 — 51 times less. This asymmetry is the central finding of Section 3 of the main paper and shows that the field has moved "from output to input"—from clinical

applications to basic biological predictions. Figure S3 supplements the heatmap from Figure 2 of the main paper with aggregate numbers for the entire ten-year period.

S3. Table S2: Overview of all queries and their results

Table S2 summarizes all 18 queries performed during the systematic search and the number of unique PMIDs returned by each query for the period 2015–2026. The numbers differ from the 365 unique papers in the overall cross-section due to two factors: (i) one paper can match multiple queries at the same time (eg the paper on AI for bioink for bone defects matches both bio_aif_bioink_design and bio_app_bone); (ii) not all papers returned by APP and AIF queries necessarily overlap with the generic BIO × AIM intersection—some are filtered by specific AIF/APP terms.

Query name	Description	Hits (2015–2026)
bio_all	All 3D bioprinting literature (baseline)	**~10.220**
aim_all	All AI literature (baseline, without bioprinting filter)	**~509.348**
bio_x_aim	**Center section: bioprinting × AI**	**365**
bio_aif_bioink_design	Section 3.1 — AI for Bioink Design	10
bio_aif_process_opt	Section 3.2 — AI for Process Optimization	27
bio_aif_monitoring	Section 3.3 — AI for real-time monitoring	38
bio_aif_viability	Section 3.4 — AI for Viability Prediction	**3**
bio_aif_scaffold	Section 3.5 — AI for scaffold/feature mapping	13
bio_aif_translation	Section 3.6 — AI for Clinical Translation	**154**
bio_app_bone	Bioprinting + AI for bones	27
bio_app_cartilage	Bioprinting + AI for cartilage	8
bio_app_skin	Bioprinting + AI for skin	19
bio_app_vascular	Bioprinting + AI for vascular structures	23
bio_app_neural	Bioprinting + AI for neural tissues	19
bio_app_organoid	Bioprinting + AI for organoid / tumor models	**90**
bio_app_liver	Bioprinting + AI for the liver	4
bio_app_cornea	Bioprinting + AI for cornea	4
bio_app_cardiac	Bioprinting + AI for cardiac	6

Background colors: blue — header; orange — central section; red — category with the least number of papers (3.4); green — APP queries.

S4. Table S3: Papers appearing in multiple AIF categories

Certain papers appear as top-cited in several queries at the same time, which indicates their **cross-domain character** — they cover more than one functional category. Table S3 shows the five most prominent such papers, with the number of AIF categories in which they appear and a brief description of the reasons for their multiple classification.

Ref.	Paper	AIF categories	Reason for multiple classification
**[18]**	*AI-driven 3D bioprinting for regenerative medicine: From bench to bedside* (Bioactive Materials, 2025)	**3.1, 3.2, 3.6**	The QbD framework links bioink design, process optimization and clinical translation.
**[7]**	*Ng et al. Progress and Opportunities for Machine Learning in Materials and Processes of Additive Manufacturing* (Adv Mater, 2024)	**3.2, 3.3**	It maps ML as an integrative axis through process control and microstructure analysis.
**[21]**	*Bonatti et al. A Deep Learning Quality Control Loop of the Extrusion-based Bioprinting Process* (Int J Bioprint, 2022)	**3.2, 3.3**	The closed-loop system combines process optimization and real-time monitoring.
**[24]**	*Tebon et al. Drug screening at single-organoid resolution via bioprinting and interferometry* (Nat Commun, 2023)	**3.3, 3.6**	Real-time monitoring of organoids for drug screening - application in clinical translation.
**[19]**	*Self-driving bioprinting laboratories* (Biofabrication, 2026)	**3.1, 3.2, 3.4**	The autonomous system combines bioink formulation, printing, and biological feedback.

Cross-domain works are a structural signal: they indicate places of integration in the field, where multiple AIF categories converge into a single system. It is particularly interesting that Self-driving bioprinting laboratories [19] includes 3.1, 3.2, and 3.4 — including 3.4, the category with the smallest number of papers in the entire dataset — which confirms the argumentation in Section 7 of the main paper that AI-native bioprinting seeks integration through all AIF categories, not through individual segments.

S5. AIF sub-axis overlap analysis

To characterize how the six AIF sub-axes relate to each other and to the generic BIO × AIM intersection, a set-theoretic overlap analysis was performed on a re-snapshot of the corpus dated 29 May 2026, using unique PMID matching rather than per-year aggregation. The script aif_overlap.py (released alongside the main pipeline) fetches the full PMID set for each of the six AIF queries and for the bio_x_aim baseline, deduplicates across years within each query, and computes set unions, intersections, and pair-wise overlaps. The May 2026 unique-PMID count for bio_x_aim is 345; the small 5–6% reduction relative to the per-year aggregated count of 365 in the 28 April 2026 data-freeze reflects PMIDs that PubMed indexes under more than one publication-date year (typically epub vs. final publication date),

which inflate per-year sums but are deduplicated under unique-PMID counting. PubMed's continued indexing growth between the two snapshots adds approximately 16 new records, which net out against the deduplication.

Across the six AIF queries, 190 unique PMIDs match at least one sub-axis; 34 papers appear in two or more sub-axes, and 5 papers appear in three or more sub-axes (the five most prominent cross-domain papers are itemized in **Table S3**). The sum of per-query unique counts (230) therefore exceeds the union (190) by 40, which is the magnitude of cross-axis double-counting. A further 155 papers (45% of the 345-paper unique intersection) match the generic bio_x_aim query without being captured by any AIF-specific search term — they discuss AI in the context of bioprinting at a methodological or conceptual level without engaging a specific pipeline function and form the long tail of the corpus.

Table S4 reports the pair-wise overlap matrix. The largest off-diagonal entries pair clinical translation with monitoring (15 shared PMIDs), process optimization (8), and scaffold-function (7); the corresponding pairing of monitoring with process optimization (8) reflects the closed-loop control architecture exemplified by Bonatti et al. [21], which simultaneously optimizes printing parameters and monitors the print online. These overlaps quantify the cross-domain character that motivates the cross-row placement convention adopted in Table 1 of the main text.

Table S4. Pair-wise overlap of AIF sub-axes (unique PMIDs shared). Diagonal entries give the per-query unique total; off-diagonal entries give the number of PMIDs shared between two sub-axes. Snapshot: 29 May 2026.

	**bioink_design**	**process_opt**	**monitoring**	**viability**	**scaffold**	**translation**
**bioink_design**	11	1	0	0	0	4
**process_opt**	1	23	8	1	1	8
**monitoring**	0	8	38	1	0	15
**viability**	0	1	1	2	0	1
**scaffold**	0	1	0	0	12	7
**translation**	4	8	15	1	7	144

The complete output of the overlap analysis (per-query PMID lists, JSON-serialized for full reproducibility) is available in the reproducibility repository as data/analysis/aif_overlap.json. The

script aif_overlap.py and the markdown summary aif_overlap.md are released alongside the main pipeline scripts.

S6. Data and code accessibility

In accordance with the principles of open science and FAIR (Findable, Accessible, Interoperable, Reusable) standards, all used material — configuration files, scripts, final data tables, and source code for figure generation — will be publicly available before publication of the paper.

S6.1 GitHub repository

The master repository will contain the complete project structure including:

- Configuration file with all search terms (config/search_terms.yaml)
- Scripts for search, citation, and analysis (Python 3, open APIs)
- Master list of works (data/top_cited/master_unique.csv) with 184 unique PMIDs
- Numerical summaries (data/analysis/numerical_summary.md)
- Source code for generating all figures from the main paper and supplementary material
- README with instructions for reproducing the complete pipeline

GitHub link: *https://github.com/zivanovicmkg/bioprinting-ai-mining-framework*

S6.2 Zenodo archive

A stable, versioned copy of the repository will be deposited on the Zenodo platform before publication of the paper, with an official DOI for scholarly citation.

Zenodo DOI: *10.5281/zenodo.19885393*

S6.3 License

All software material will be released under the MIT License; all data under CC-BY-4.0 license. This choice of licenses allows unconditional sharing and adaptation, with mandatory citation of the source.

S6.4 Correspondence

For questions related to methodology, data or code, the corresponding author is Marko N. Živanović (University of Kragujevac, marko.zivanovic@uni.kg.ac.rs).

— End of Supplementary Materials —
